# American Medical Association

**Published:** 1859-03

**Authors:** 


AMERICAN MEDICAL ASSOCIATION.—We would remind our readers that the Twelfth Annual Meeting of the Association will be held on the 3d of May, 1859, at Louisville, Kentucky. The Secretary, Dr. A. M. Bemiss, desires that all bodies represented in the meeting will send him a correct list of their delegates so soon as appointed. The convention of professors from the different medical schools throughout the country will meet at the same place the day before the sitting of the Association.

